# The role of community social capital in the relationship between socioeconomic status and adolescent life satisfaction: mediating or moderating? Evidence from Czech data

**DOI:** 10.1186/s12939-016-0490-x

**Published:** 2016-12-12

**Authors:** Thomas Buijs, Lea Maes, Ferdinand Salonna, Joris Van Damme, Anne Hublet, Vladimir Kebza, Caroline Costongs, Candace Currie, Bart De Clercq

**Affiliations:** 1 Unit Health Promotion, Department of Public Health, Ghent University, De Pintelaan 185, K3, B-9000 Ghent, Belgium; 2 Institute of Active Lifestyle, Faculty of Physical Culture, Palacky University in Olomouc, Tr. Miru 15, Olomouc, 77111 Czech Republic; 3 Department of Psychology, Faculty of Arts, Charles University in Prague, Ovocný trh 3-5, 116 36 Praha 1, Czech Republic; 4 EuroHealthNet, 67 rue de la Loi B-1040, Brussels, Belgium; 5 School of Medicine, Medical & Biological Sciences, University of St. Andrews, North Haugh, St Andrews, KY16 9TF UK

**Keywords:** Health inequalities, Social capital, Life satisfaction, Youth, Czech Republic

## Abstract

**Background:**

The concept of social capital has been extensively used to explain the relationship between socioeconomic status (SES) and adolescent health and well-being. Much less is known about the specific mechanism through which social capital impacts the relationship. This paper investigates whether an individual’s perception of community social capital moderates or mediates the association between SES and life satisfaction.

**Methods:**

This study employs cross-sectional data from the 2009–2010 Czech Health Behaviour in School-Aged Children survey: a WHO Collaborative Cross-National Study (HBSC). A sample of 4425 adolescents from the 5^th^, 7^th^ and 9^th^ grade (94.5% school response rate, 87% student response) was used to perform multilevel analysis.

**Results:**

We found that pupils’ life satisfaction was positively related to both family affluence and perceived wealth. Moreover, we found the cognitive component of social capital to be positively associated with life satisfaction. Additionally, a significant interaction was found, such that the social gradient in life satisfaction was flattened when pupils reported high levels of perceived community social capital.

**Conclusions:**

The present findings suggest that community social capital acts as an unequal health resource for adolescents, but could potentially represent opportunities for public health policy to close the gap in socioeconomic disparities.

## Background

Social inequalities are an important public health topic concerning the entire population [1]. Differences in health outcomes do not only exist between the lowest and highest socioeconomic classes, but follow a gradient pattern [2, 3]. Regarding adolescents, a large body of evidence documents the relationship between social inequalities and health [4]. Moreover, recent time-series analysis has shown that socioeconomic inequalities in adolescent health have increased from 2002 to 2010 in 34 North American and European countries [5]. Targeting young people and their communities is fundamental because (1) social inequalities pass across generations [1] and (2) what happens throughout childhood is likely to have a life-long impact [6] since virtually every aspect of early human development (physical, cognitive, and socio-emotional) is sensitive to external influences [4, 7, 8]. Traditional policy interventions, focusing on individuals’ attributes, knowledge and skills have been shown to merely produce limited effects [9], whereas ample research on the other hand has shown that young people’s health and well-being is shaped, not only by personal decisions, but also by the routine organization of everyday settings like neighborhoods, communities, schools and other social networks [10–12]. It is in this context that social capital emerged as a prominent concept in public health to explain the relationship between socioeconomic status (SES) and people’s health and well-being and to suggest policy options [13]. This has led to the incorporation of social capital into the WHO’s general conceptual framework on the social determinants of health [14]. Social capital and initiatives that increase cohesion, co-operation and interpersonal trust, can play an important role in decreasing socioeconomic inequalities between adolescents with low and high SES, thus effectively flattening the social gradient. Evidence shows that health gains incurred by increasing social capital are particularly marked for disadvantaged (or vulnerable) children and young people in communities with low social capital [15, 16]. Much less is known however on the specific mechanism, i.e. mediating versus moderating, through which social capital operates. Nevertheless, this is a crucial aspect for policy makers in order to transform existing research into policy. Moreover, any discussion on social inequalities in health and social capital has to be framed within the concept of the welfare regime theory [17]. As such, analyzing Czech data provides a unique case since research in Central and Eastern European welfare countries remains limited to date.

### Social capital: conceptual review

Since its introduction, social capital has been variously defined, from being an individual resource embedded in social relations [18, 19] to “features of social organization such as networks, norms, and social trust that facilitate coordination and cooperation for mutual benefit” ([20], p.67). Orthogonal to the distinction between “individual” and “collective” social capital, most conceptualizations can be decomposed into a *structural* and a *cognitive* component [21, 22]. Structural social capital refers to objectively measurable characteristics such as participation in clubs, neighborhood activities and other social networks. Cognitive social capital refers to the perception of level of trust and reciprocity, through norms, values and attitudes. Within the structural component of social capital, an important distinction is made between bonding, bridging and linking components, which respectively refer to connections between people of similar social groups, different social groups and links with external sources of power [23, 24].

Despite the lack of agreement in terms of a clear definition for social capital more than three decades after the reintroduction of social capital by Bourdieu, Coleman and Putnam, it is hard not to be impressed with the overwhelming evidence indicating that social capital is an important determinant of major health outcomes (e.g. [17, 24–32]).

#### Life satisfaction, health and social capital

Subjective well-being (SWB) is an important topic within public health research. A growing body of research evidence shows significant associations between high subjective well-being and positive health outcomes such as positive adolescent development [33–35], decreased suicide rates [36] and increased health and longevity [37]. Research in the field of positive psychology recognizes three distinct categories that compose SWB: emotional responses (positive affect: joy, optimism, negative affect: sadness, anger), domain satisfactions (such as work satisfaction or relationship satisfaction) and life satisfaction [38], all three of which are commonly used to measure SWB interchangeably. Though little empirical research has been conducted to differentiate these heterogeneous aspects of SWB thus far, scientific consensus is that life satisfaction, i.e. a subjective evaluation of the overall quality of life, is the most stable indicator and therefore the key indicator when studying links between SWB and health outcomes [37]. The concept of life satisfaction is of specific interest when studying inequalities in adolescent health, considering the formative nature of this life stage and thus its significant impact on health outcomes later in life [4]. Research has shown clear links between life satisfaction and physical activity [39, 40], substance use [41–43] and unhealthy diet [44]. Moreover, previous research has shown that life satisfaction is unevenly distributed between adolescents with low and high SES [45, 46]. As such, researching social capital in relation to adolescent life satisfaction is meaningful in order to identify opportunities for policy makers to reduce socioeconomic inequalities and improve the health of low SES adolescents. Recently, several studies have investigated the cross-sectional relationship between social capital and life satisfaction using the Cantrill ladder [47], a reliable and valid instrument, using relatively simple self-rating questions about life satisfaction and more generally, happiness [48–50]. Most research studies so far have found a positive relationship between social capital and life satisfaction [12, 51, 52].

In the context of community social capital, which is analyzed in this paper, the preponderance of the evidence suggests that adolescents with a wider range of (or higher quality of) social support networks, benefit through better general health, quality of life and life satisfaction. The most complete literature review to date included 39 research papers that studied the effect of community social capital on life satisfaction; 26 studies showed a positive relation and 13 showed no relation, whereas not a single study found a negative relation [12]. Specifically school [16, 53–55] and neighborhood environments [15, 56, 57] were found to be beneficial in promoting better outcomes.

#### Mediating/moderating: empirical evidence

Limited research is available on the nature of the social capital mechanism, that is to say whether social capital moderates or mediates the relationship between socioeconomic position and life satisfaction. Nevertheless, the mechanism through which community social capital influences life satisfaction is highly important for policy makers. A mediating, or indirect relation, implies that a third variable underlies an observed relationship between two variables [58]. This would mean that high SES leads to higher community social capital, which would in turn lead to higher life satisfaction. A moderating, or direct relationship, on the other hand, implies that a third variable directly influences the relationship between two other variables [58, 59]. This would mean that community social capital directly impacts the relation between SES and life satisfaction. It is obvious that the latter scenario is to be preferred by policy makers as it would allow them to employ community social capital as a health resource.

Moreover, most of the available research focuses on health, rather than well-being. In an individual-level study on the psychosocial pathway of health inequalities, Veenstra [60] found only little evidence for the individual effects of social capital on self-rated health status and no evidence for a mediating mechanism. However, these findings should be treated with caution given some serious limitations such as a small sample size (*n* = 534) and low response rate (40%). After a decade of social capital research, a similar study was set up by Dahl [61] hypothesizing that individual social capital may mediate the impact of socioeconomic position on health. However, the results of the study did not confirm this expectation for health outcomes as perceived health and longstanding illness. In contrast to this finding, the results of Lindström et al. [62] support the idea that social capital is an important mediating link behind the socioeconomic differences in leisure-time physical activity and, ultimately, cardiovascular diseases. Few studies on social capital and health inequalities studied outcomes in children. In a recent review on neighborhood social capital and the gradient in adolescent health, Vyncke et al. [63] found a total of eight studies, of which just two found evidence for a mediating social capital mechanism for respectively mental health problems [64] and verbal ability and behavioral problems [65]. Since the evidence for a mediating mechanism of social capital in the individual relationship between socioeconomic position and (child) health, is inconclusive, the present study hypothesizes that the psychosocial mechanism of social capital may be a moderating one. In line with others who demonstrated a moderating social capital mechanism for internalizing and externalizing behavioral [66] and antisocial behavioral problems [67]. In a sample of young children and adolescents, the authors already found evidence for a moderating effect of community social capital at the contextual level on adolescent perceived health and wellbeing [15]. Others [68] also found evidence for a moderating effect of community social capital at the individual level for a variety of outcomes. They demonstrated that social capital nullified SES effects on psychological symptoms and life satisfaction and narrowed SES differences in somatic symptoms, injuries and fighting. Similar findings were reported for community social capital at the contextual level [15].

#### Welfare regime theory: a brief introduction

The protective effect of social capital on health inequalities may not be invariant for national contexts since different welfare regimes may modify the impact of both social inequality [69, 70] and social capital [17] (see Eikemo and Bambra [71] for an introduction to welfare regime theory). More specifically for adolescents, Richter et al. [72] found that regime type contributed to the explanation of cross-national variations in adolescent self-rated health and health complaints, but for social inequalities in health they could not find a clear pattern between welfare regime types. Regarding to social capital, Islam et al. [17] argue that the influence of social capital might be less salient in egalitarian countries (defined by comparatively low levels of income inequality and strong welfare states). Yet, recent studies in typical egalitarian countries like Norway [73] and Japan [74] reveal substantial differences in social capital and associations with health outcomes. In general, the protective effect of social capital on health inequalities was examined mainly in *Anglo-Saxon (liberal)* countries (e.g. [60, 66–68]), and more recently also in countries representing the *Bismarckian (conservative)* (e.g. [15]) and *Scandinavian (social democratic)* welfare regime types (e.g. [61]), but much less is known about the situation in *Eastern* and Central European welfare countries such as Slovakia, Poland, Hungary and the Czech Republic. After the breakdown of their communist regimes they are still undergoing extensive social reforms and should be treated as a distinctive regime type [75].

#### Aim

The objectives of this research are to investigate whether an individual perception of community social capital moderates or mediates the association between SES and adolescent well-being. Life satisfaction was used as outcome measure [47]. In accordance with Dahl [61], we conceptualize community social capital as it empirically appears at the individual level only. In addition, the present study investigates data from the Czech Republic being a unique case for the interrelationships between social inequality and social capital because (1) evidence suggests that social capital plays a comparatively smaller role in egalitarian countries [17] and (2) despite similar Gini coefficients^1^ [76] the historical and socio-political context is significantly different from other egalitarian countries characterized by the well-known Nordic welfare model [77–81].

## Methods

### Sample

Cross-sectional data from the 2009–2010 survey of Czech Health Behaviour among School-aged Children, was used. This survey is part of the international Health Behaviour in School-Aged Children survey: a WHO Collaborative Cross-National Study (HBSC) [82]. The aim of the HBSC study is to describe young people’s health and health behavior, and to analyze how these outcomes are associated with social contexts. Self-completion questionnaires were administered in school classrooms, with requirements in terms of sampling, questionnaire items and survey administration being set out in a standardized research protocol [83]. All of the questions used in the HBSC survey must have evidence of reliability and validity when used in multiple countries before they are considered for inclusion. From a list of schools, based on information from the Institute for Information on Education, a contributory organization of Ministry of Education, Youth and Sport, 91 schools from all 14 regions of the Czech Republic were randomly selected to create a representative sample. 86 schools took part in the survey, representing a 94.5% school response rate. According to the protocol of the HBSC study classes from the 5th to 9th grades were selected randomly, one from each grade per school. According to the international research protocol, set forward by HBSC [82], data were obtained from 5284 adolescents from the 5th, 7th and 9th grade of elementary schools in the Czech Republic (response: 87%). Non-response due to absence was 13%.

Participation in the study was fully voluntary and anonymous with no explicit incentives provided for participation. The questionnaires were administrated by trained research assistants in the absence of a teacher during regular class time. Parents were informed about the study from the school administration and could opt out if they disagreed. Data collection was done under the principles of the Declaration of Helsinki and legal and regulatory requirements applicable in the Czech Republic.

For the purpose of this paper adolescents of 11 years (*n* = 1426), 13 years (*n* = 1456), 15 years (*n* = 1522) are included. The final sample consists of 4425 Czech pupils (49% boys).

### Dependent variable

Life satisfaction is measured by the following question: ‘How happy would you say you are with your life?’ [47]. This variable consists of a discretionary 10-item numerical response ladder (range 1 = worst possible life – 10 = best possible life).

### Independent variables

This study applies two different indicators for SES, both reflecting different aspects: Family affluence is an indicator of *absolute wealth* [84]. The family affluence scale (FAS) is a composite indicator of self-reported socioeconomic status comprising four items that address family assets or conditions that indicate material wealth: ‘Does your family own a car, van or truck? (0 = no; 1 = yes one; 2 = yes two or more); Do you have your own bedroom? (0 = no; 1 = yes); During the past 12 months, how many times did you go on holiday with your family? (0 = not at all, 1 = once, 2 = twice, 3 = more than twice); How many computers does your family own?’ (0 = none, 1 = one, 2 = two, 3 = more than two). Responses are summed on a 1 to 10 scale with higher scores indicating greater affluence. From its early development, there have been efforts to validate the family affluence scale at both national and international levels [85]. There is a strong agreement between children’s reports on family affluence scale items and their parents’ report [86].

The perceived wealth indicator reflects the concept of *relative wealth* and is measured using the following question: ‘How well off do you think your family is?’ (0 = not at all well off; 1 = not very well off; 2 = average; 3 = quite well off; 4 = very well off).

Measures of social capital, measured at the individual level, are employed, using the decomposition between a structural and a cognitive component (Baum & Ziersch [21]; Harpham et al., [22]). Structural social capital is measured by the participation in clubs: ‘Are you involved in any of these kinds of clubs or organizations?’ Response categories: sports club, voluntary service, political organization, cultural organization, church or religious group, youth club, other club (0 = no, 1 = yes). An unweighted sum score was calculated (range 0 – 7). Cognitive social capital is measured using a 5-item scale [87]: ‘People say ‘hello’ and often stop to talk to each other in the street; it is safe for younger children to play outside during the day; you can trust people around here; there are good places to spend your free time; I could ask for help or a favor from neighbors (1 = strongly disagree, 2 = disagree, 3 = neither agree nor disagree, 4 = agree, 5 = strongly agree). An exploratory factor analysis was performed, using Mplus 7.11 [88] to determine whether all included items load onto a unique factor or not. The EFA entails that an oblique rotation of factors was performed allowing factors to co-vary. Values below 0.05 on the Root Mean Square Error of Approximation (RMSEA) and a value of 0.90 or greater on the Comparative Fit Index (CFI) were considered as indicative of a good fit. The results showed that all items load onto a unique factor (results available from the authors).

### Analysis

Multilevel modeling is employed using ML*wi*N software (version 2.32) to account for the hierarchical structure of the data, i.e. non-random clustering of pupils in classes and schools [89, 90]. All models are three-level random intercept models including variables on level 1 only and with no random slopes. Level 1 units in our sample are pupils (*n* = 4425), level 2 units are classes (*n* = 246), and level 3 units are schools (*n* = 86). Models are calibrated using the Maximum Likelihood procedure which utilizes the (Restricted) Iterative Generalized Least Squares algorithm [91].

First, an empty model (intercept-only) with no explanatory variables was estimated in order to decompose the variance of the intercept into variance components for each of the three levels. The variation by class and school was expressed as intraclass correlation coefficient (ICC). This model is useful as a null model that serves as a benchmark with which other models are compared (Model 1). Model 2 includes the socio-demographic variables as covariates. In a third step, a model with individual socioeconomic predictors, namely, family affluence *(absolute indicator)* and perceived wealth *(relative indicator)* is conducted (Model 3). Structural social capital and cognitive social capital are added as explanatory variables in Model 4. This model estimates the contribution of both the structural and the cognitive component of social capital to life satisfaction.

In order to assess the *moderating* association between social capital and social inequalities in health, a similar approach as De Clercq et al. [15] is used. Interaction terms are estimated between both SES indicators and both components of social capital (Model 5a-d). To test the improvement of fit for each model, −2log-likelihood deviance values are calculated [90]. The conventional 5% level is used to determine statistical significance. In order to measure if social capital provides a mediating mechanism between SES and life satisfaction, the product a x b was tested for significance using bootstrapped standard errors. This product covers the effect of SES (i.e. family affluence or perceived wealth) on a mediator (i.e., structural or cognitive component of social capital) multiplied by the effect of a mediator on life satisfaction, controlled for SES [92]. Significance was tested using the Sobel test [93].

## Results

### Descriptive results

Table 1 includes descriptive statistics of the variables used in the analysis. Respondents (*n* = 4425) are divided into three age groups (11, 13 and 15 years old) and hierarchically clustered within classes (*n* = 246) and schools (*n* = 86). Both boys and girls are proportionally distributed in the overall sample.

**Table 1:** Descriptive statistics over variables related to socio-demographic background, socioeconomic status, social capital and life satisfaction

Socio-demographics		
Gender		
Boy	%	48.5
Girl	%	51.5
Age		
11	%	32.2
13	%	32.9
15	%	34.4
Socio-economics		
Family affluence	Mean-mode (s.d.), range = 1 - 10	6.32-6 (1.75)
Perceived wealth	Mean-mode (s.d.), range = 1 - 5	3.33-3 (0.78)
Social capital		
Structural social capital	mean-mode (s.d.), range = 0 - 7	1.40-1 (1.26)
Cognitive social capital	mean-mode (s.d.), range = 1 - 5	3.69-3 (0.68)
Life satisfaction	mean (s.d.), range = 1 - 10	7.49 (1.84)

### Multivariate associations: SES, social capital and life satisfaction

Table 2 presents the results of four successive multilevel models. The first model shows that 3,7% of the observed variance in life satisfaction is at the class level while no variance is observed at the school level. The grand mean is 7.499 (range 1 – 10). This is the average life satisfaction for all respondents (*n* = 4425) within all classes (*n* = 246) and schools (*n* = 86). The second model shows that the socio-demographic variables are significantly associated with pupils’ life satisfaction: girls and older adolescents report lower levels of life satisfaction (*p* < 0.01). After taking into account these individual socio-demographic factors, the amount of class variance was significantly reduced: about half of the class level variance in life satisfaction is attributable to the gender and age composition of the classes. Model 3 accounts for the SES indicators. We observe a significant social gradient in the relationship between socioeconomic position and life satisfaction. More precisely, pupils’ life satisfaction is positively related to both family affluence and perceived wealth (*p* < 0.01), but the *relative* indicator (perceived wealth) is a much stronger determinant for life satisfaction than the *absolute* indicator (family affluence). Adding the SES indicators turned the effect of gender non-significant. In model 4 we added the social capital variables. Only the cognitive component of social capital is positively associated with life satisfaction (*p* < 0.01). There is no statistically significant effect of structural social capital. Overall, the associations in the fourth model did not substantially change from the third model. Moreover, the effect of cognitive social capital did not explain away the relationship between SES indicators and life satisfaction.

**Table 2:** Fixed and random parameters of the three-level life satisfaction model

	Model 1		Model 2		Model 3		Model 4	
	B	(S.E.)	B	(S.E.)	B	(S.E.)	B	(S.E.)
Fixed effects								
Constant	7.499	(0.036)***	7.900	(0.064)***	7.799	(0.063)***	7.725	(0.060)***
Individual-level								
Socio-demographics								
Female			−0.201	(0.056)**	−0.092	(0.054)^n.s.^	−0.073	(0.053)^n.s.^
Age (ref: 11)								
13			−0.312	(0.079)**	−0.295	(0.077)**	−0.245	(0.073)**
15			−0.548	(0.078)**	−0.439	(0.076)**	−0.304	(0.074)**
Socioeconomics								
Family affluence					0.096	(0.016)**	0.077	(0.016)**
Perceived wealth					0.538	(0.037)**	0.446	(0.036)**
Social capital								
Structural social capital							0.033	(0.021)^n.s.^
Cognitive social capital							0.689	(0.041)**
Class-level								
-								
School-level								
-								
Random effects								
Individual-level variance	3.253	(0.072)***	3.250	(0.073)***	3.002	(0.067)***	2.813	(0.064)***
Class-level variance	0.124	(0.028)**	0.058	(0.028)*	0.057	(0.026)*	0.047	(0.024)*
School-level variance	0.000	(0.000)^n.s.^	0.009	(0.018)^n.s.^	0.011	(0.017)^n.s.^	0.005	(0.015)^n.s.^
Log likelihood	17281.1		17156.0		16611.4		16112.9	
Δ Log likelihood (Δ df)			125.1 (3)		544.6 (2)		489.5 (2)	
* p*			< .001		< .001		< .001	

Figures in parentheses represent standard errors

*n.s.* not significant

*p < 0,05, **p < 0,01, ***p < 0,001

### Interaction terms: Does social capital modify social inequalities in life satisfaction ?

**Fig. 1:**
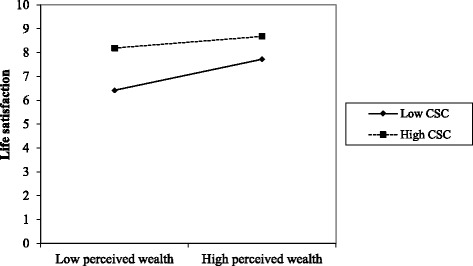
Predicted relationship between perceived wealth and life satisfaction for low and high level of community social capital (CSC) based on the fixed part results from Model 5d (*p* < 0.01)

Table 3 presents the results of four multilevel models that investigate all possible interactions between both the relative and absolute SES measures and both components of social capital. Model 5a-c were not interpretable since their deviance statistic was not significant, indicating that these models did not fit the data better than the more simple model 4. The final model 5d examines the interaction between perceived wealth and cognitive social capital (*p* < 0.01). Figure 1 plots the predicted relationship between perceived wealth and life satisfaction for low and high levels of cognitive social capital, indicating that the social gradient in life satisfaction was leveled-up when pupils reported a high level of cognitive social capital.

**Table 3:** Interaction terms of the life satisfaction model

	Model 5a		Model 5b		Model 5c		Model 5d	
	B	(S.E.)	B	(S.E.)	B	(S.E.)	B	(S.E.)
Fixed effects								
Family affluence x structural social capital	0.003	(0.011)	-		-		-	
Family affluence x cognitive social capital			−0.017	(0.022)	-		-	
Perceived wealth x structural social capital					0.037	(0.025)	-	
Perceived wealth x cognitive social capital							−0.204	(0.045)**
Random effects								
Individual-level variance	2.813	(0.064)	2.813	(0.064)	2.811	(0.064)	2.799	(0.063)***
Class-level variance	0.048	(0.024)	0.047	(0.024)	0.047	(0.024)	0.046	(0.024)^n.s^
School-level variance	0.005	(0.015)	0.005	(0.015)	0.005	(0.015)	0.007	(0.015)^n.s^
Log likelihood	16112.8		16112.3		16110.6		16092.1	
Δ Log likelihood (Δ df)	0.1 (1)		0.6 (1)		2.3 (1)		20.8 (1)	
*p*	0.751		0.438		0.219		<.001	

Figures inn parentheses represent standard errors

Model 4 was the control and reference model for Δ Log likelihood (Δ df) calculations of Model 5a - d

*n.s.* not significant

**p < 0,01, ***p < 0,001

Looking for a potential mediating effect, we observed a significant direct relation between family affluence and perceived wealth, and life satisfaction (Table 2, Model 3). Both, family affluence and perceived wealth significantly affected structural and cognitive social capital (a-path) (Table 4), which in turn significantly affected life satisfaction (b-path) (Table 2, Model 4). Looking at the indirect effects, only cognitive social capital was found to be a significant mediator of both relations between SES and life satisfaction (Table 4).

**Table 4:** Indirect effects and a-paths of the life satisfaction model

	ab-path		a-path	
	B	(S.E.)	B	(S.E.)
Family affluence > structural social capital > life satisfaction	0.003	(0.002)^n.s.^	0.080	(0.011)***
Family affluence > cognitive social capital > life satisfaction	0.014	(0.004)***	0.020	(0.006)***
Perceived wealth > structural social capital > life satisfaction	0.003	(0.002)^n.s.^	0.083	(0.026)**
Perceived wealth > cognitive social capital > life satisfaction	0.100	(0.011)***	0.145	(0.013)***

Figures in parentheses represent standard errors

Analyses are based on Model 4: b-paths can be found under this model

*n.s.* not significant

**p < 0.01,***p < 0,001

## Discussion

In the last fifteen years researchers and policy makers have paid increasing attention to the concept of social capital as a major topic for public health [94–96]. The aim of the present study was to investigate whether an individual perception of community social capital moderates or mediates the association between SES and adolescent life satisfaction. Previous studies have demonstrated a moderating mechanism both at the contextual [15, 66, 67] and individual level [68].

The results from the multivariate interaction models partly confirmed this expectation. Only one of the four interaction models (Model 5d) showed significant fit to the data, indicating that the association between perceived wealth and life satisfaction was moderated by cognitive social capital. Research looking at a mediating mechanism has been mixed with most studies showing little evidence [60–62]. However, in this study results from the Sobel test showed that cognitive social capital is a significant mediator in the relationship between life satisfaction and both FAS and perceived wealth.

Our findings add to the existing knowledge in five ways. Firstly, the study differentiated between the two main components of social capital [21, 22]: the structural component and the cognitive component. Previous studies emphasized the importance of this differentiation since each component may be associated differently with health and well-being [97, 98]. Results from our mutually adjusted model (Model 4) showed that cognitive social capital was positively and independently associated with adolescent life satisfaction while the association with structural social capital was not significant. Although not entirely in line with previous research, the absence of a significant association between the structural component of social capital and adolescent life satisfaction does not come as a complete surprise for two reasons. Firstly, our results confirm a more recent study that investigated the relationship between social capital and self-rated wellbeing in Europe. While measures of structural social capital were significant for the full sample, decomposition into geographical subgroups showed that this association was mainly driven by Western-Europe, whereas the relationship was much weaker in Eastern-Europe (including the Czech Republic), where several of the indicators were insignificant [99]. The authors concluded that it seems as if local culture, social institutions, customs and traditions drive the strength of social capital linkages to life satisfaction in different parts of Europe. More research is needed however to confirm this claim. More broadly, the lack of association between structural social capital and adolescent life satisfaction can be viewed in the ongoing debate on the importance of absolute affluence versus relative inequality. Previous research has indicated that as countries reach a certain GNP per capita (typically 10,000-13,000), the effect of relative inequality becomes increasingly important [100, 101]. Recent research, using HBSC data, has also been shown to be in line with this hypothesis [102]. Secondly, the present study offered some evidence to support the validity of the social cohesion and collective social pathway theory [103], linking individual socioeconomic status (SES) to well-being. More specifically, we found evidence for both the moderating and the mediating variant. This finding is in line with Elgar et al. [68], but contradicts the results found in Dahl et al. [61]. Thirdly, a moderating effect of social capital opens new perspectives for health promotion since such a mechanism implies that social capital may be a way to decrease socioeconomic inequalities between adolescents with low and high SES, thus effectively flattening the social gradient in health. A mediating mechanism would imply that one’s socioeconomic position affects the availability and allocation of social capital as a health asset. In contrast, a moderating mechanism allows people to address health assets regardless of their personal characteristics. Note that neo-materialist explanations will still consider this as a ‘workaround solution’, arguing that interpretations should start with the structural and material causes of inequalities rather than with perceptions of inequality [104]. Either way, our findings emphasize the need for more research on mediating and moderating mechanisms in order to understand the relation between SES and young people’s life satisfaction. Fourthly, the present results are discussed in the light of the welfare regime theory [69, 70]. Of course, only comparative studies can make statements about variability in social inequality and social capital between countries, but our results do indicate that both matter in the Czech context: (1) relative and absolute SES indicators were both positively and independently related to adolescent life satisfaction, and (2) cognitive social capital was positively related to adolescent life satisfaction. The findings on social inequality in adolescent life satisfaction were not surprising. Mackenbach [105] reviews nine theories that may potentially explain such persisting and even widening social inequalities. The author concludes that the psychosocial theory cannot explain the widening of social inequalities, but may explain why social inequalities persist. Despite the welfare state, considerable differences in power and prestige have continued to exist. Some of the inequalities in psychosocial stress may have blurred, but the middle classes have also benefited from the welfare state, for example because it has reduced the psychosocial stress of job insecurity even among the employed [106]. Moreover, advanced welfare states may raise unrealistic expectations of a better life among people with a lower socioeconomic position, and therefore induce higher levels of frustration and stress [107, 108].

Finally, social capital in Eastern Europe has received a fair amount of scholarly attention, also in the Czech Republic [109]. Fidrmuc and Gerxhani [110] conclude that the low average stock of social capital in Central and Eastern European countries can be attributed to the lower level of economic development and the lower quality of institutions in the new member countries. The study compared levels of social capital in the Czech Republic with average levels of both old and new EU member states and with neighboring states. Slovakia (which together with the Czech Republic formed Czechoslovakia until its dissolution in 1993), Poland and East Germany represented the “post-communist countries”, whereas West-Germany and Austria were labeled as “old EU member states”. They found that social capital in the Czech Republic (measured by a large set of participation indicators) seems to be closer to the average values of the old EU member states than to the new member states. On the other hand, the generalized social trust value from the 1990 and 1999 World Values Survey were closer to the values of other Eastern European countries than to Western ones. The discrepancy between structural and social capital appears to be an indicator of two important aspects of the Czech society: (1) the fact that structural social capital is closer to the levels observed in longstanding EU member states could possibly reflect the Czech Republic’s status as one of the most developed former Eastern bloc countries in economic terms. (2) The low stock of cognitive social capital however is hypothesized to be indicative of the corruption (a form of negative social capital), which was found to be higher than the economic performance would suggest [111]. Fidrmuc and Gerxhani [110] found the same pattern for different measures of altruism. Previous results concerning the interrelationships between cognitive social capital and health inequalities in the Czech Republic did not support any major differences with other Anglo-Saxon (e.g. [60, 66–68]) and Bismarckian countries (e.g. [15]).

### Weaknesses

Despite the use of hierarchical multilevel modeling techniques, we did not control for the contextual dimension of social capital. This is problematic for several reasons. First, several key authors [112, 113] stress the cross-level nature of area or place effects in multilevel investigations on social capital and population health and by extension contextual effects in social epidemiology [114]. Confounding from compositional factors, i.e. to separate the influence from individual characteristics of the people who live in certain communities and characteristics of the communities, is of major importance in studies of contextual associations [115]. This problem is especially pertinent to measures of cognitive social capital [112], whereby the contextual variable is the aggregated version of the individual responses [116]. Second, the present analytical approach does not add to the theoretical discussion regarding the level of aggregation of social capital – both for structural and cognitive components – in relation to health and well-being outcomes (e.g. [112, 117–120]). Third, the current analytical treatment of social capital limits the comparability with other work on social capital and health inequalities that considered multiple levels of aggregation (e.g. [15, 67]). Another weakness is the limited generalizability of the results given the specific (cultural) context of the Czech Republic. Moreover, this study applied two measures to capture socioeconomic status, i.e. FAS and relative family wealth. Current scientific evidence acknowledges the complexity of the SES construct and recognizes that a multiple measure approach is required to capture its full complexity [121–123]. Nevertheless, what this approach should look like has so far not been identified and as such, the current approach is quite likely suboptimal.

### Strengths

The most important strength of the study was the introduction of a unique dataset. In general, the understanding of social capital in Eastern Post-communist countries is largely focused on participation levels and institutional measures in relation to corruption [110]. Very little is known about social capital among young people. As the previous Czech HBSC surveys implemented in 1994, 1998, 2002, and 2006 did not include measures of social capital, the 2010 survey was the first opportunity to perform analyses.

## Conclusion

Young peoples’ experiences can impact their well-being and have long-term consequences [6]. Positive exposures in early life can bolster a child and young person’s long-term well-being, and help them build a ‘capital reserve’ that can be of benefit throughout life, whereas negative exposures can undermine this. The present findings suggest that cognitive social capital acts as an unequal health resource for adolescents, but could potentially represent opportunities for public health policy to close the gap in socioeconomic disparities.

## Abbreviations

CFI: Comparative fit index
EU: European Union
FAS: Family affluence scale
HBSC: Health Behaviour in School-aged Children
ICC: Intraclass correlation coefficient
RMSEA: Root Mean Square Error of Approximation
SES: Socioeconomic status
SWB: Subjective well-being
WHO: World Health Organization

## Footnotes

1 Gini coefficients for the Czech Republic, Denmark, Finland, Norway, and Sweden were respectively 0.26, 0.25, 0.26, 0.25 and 0.26.
